# *N*-hexanoyl chitosan stabilized magnetic nanoparticles: Implication for cellular labeling and magnetic resonance imaging

**DOI:** 10.1186/1477-3155-6-1

**Published:** 2008-01-04

**Authors:** Shanta R Bhattarai, Remant B Kc, Sun Y Kim, Manju Sharma, Myung S Khil, Pyoung H Hwang, Gyung H Chung, Hak Y Kim

**Affiliations:** 1 Department of Pharmaceutical Sciences, Wayne State University, Detroit, MI, 48202 USA; 2 Department of Bionanosystem Engineering, Chonbuk National Univiversity, Jeonju, South Korea; 3 Deparrtment of Pediatric and Clinical Research Center, School of Medicine, Chonbuk National Univiversity, Jeonju, South Korea; 4 Department of Textile Engineering, Chonbuk National Univiversity, Jeonju, South Korea; 5 Department of Radiology, School of Medicine, Chonbuk National Univiversity, Jeonju, South Korea

## Abstract

This project involved the synthesis of *N*-hexanoyl chitosan or simply modified chitosan (MC) stabilized iron oxide nanoparticles (MC-IOPs) and the biological evaluation of MC-IOPs. IOPs containing MC were prepared using conventional methods, and the extent of cell uptake was evaluated using mouse macrophages cell line (RAW cells). MC-IOPs were found to rapidly associate with the RAW cells, and saturation was typically reached within the 24 h of incubation at 37°C. Nearly 8.53 ± 0.31 pg iron/cell were bound or internalized at saturation. From these results, we conclude that MC-IOPs effectively deliver into RAW cells *in vitro *and we also hope MC-IOPs can be used for MRI enhancing agents in biomedical fields.

## Background

Magnetic particles ranging from the nanometer to micrometer scale are being used in an increasing number of medical applications. The important properties of magnetic particles for medical applications are nontoxicity, biocompatibility, injectability, and high level accumulation in the target tissue or organ; the most important property among those mentioned above is nontoxicity. Magnetic nanoparticles offer attractive and versatile applications in the field of biotechnology, such as DNA and RNA separation, cell separation, drug delivery system (DDS), magnetic resonance imaging (MRI), and hyperthermia [1-6]. For these applications, magnetic iron oxides such as Fe_3_O_4 _or gamma-Fe_2_O_3 _are employed as a magnetic phase because they are stable and harmless to the living bodies. To make them bind to a biological entity, their surfaces are usually modified with an appropriate compound such as polyethyleneglycol (PEG) or streptavidin. Polymers like poly-L-lysine (PLL), poly ethylene imide (PEI) and dextran, and recently chitosan [6] has been used as a stabilizer (coating agent) for iron oxide nanoparticles so as to improve the nanoparticle's biocompatibility and injectability. However, high-level accumulation in the target tissue or organ and cytotoxicity; the most important property of the nanoparticles is remains to be intact.

More or less to improve limitations stated above, several derivatives of chitosan have been proposed based on reactions with the free amino groups. Our research group already investigated the hydrophobic modification of natural chitosan by using three different acyl chlorides (hexanoyl, octanoyl and myristoyl chloride) so as to improve its aqueous solubility and subsequently used them for stabilization of metalic nanoparticles [7-9]. In this paper, we have selected the hexanoyl chloride modified chitosan stabilized iron oxide nanoparticles (*N*ac-6-IOPs or simply MC-IOPs) as a material of interest and demonstrated its biomedical application like cellular labeling, and MRI using mouse macrophages cell line (RAW cells).

## Results and discussion

### Synthesis and characterization of MC-IOPs

**Figure 1:**
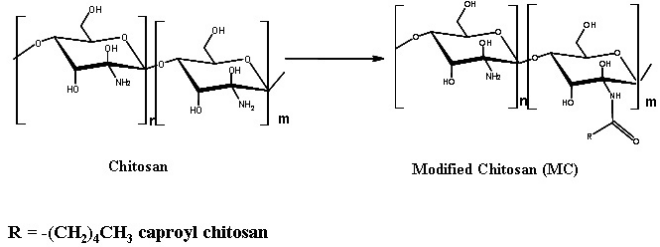
Chemical structure of chitosan and modified chitosan.

The chemical structure of the native and modified chitosan is shown in Figure 1.

**Figure 2:**
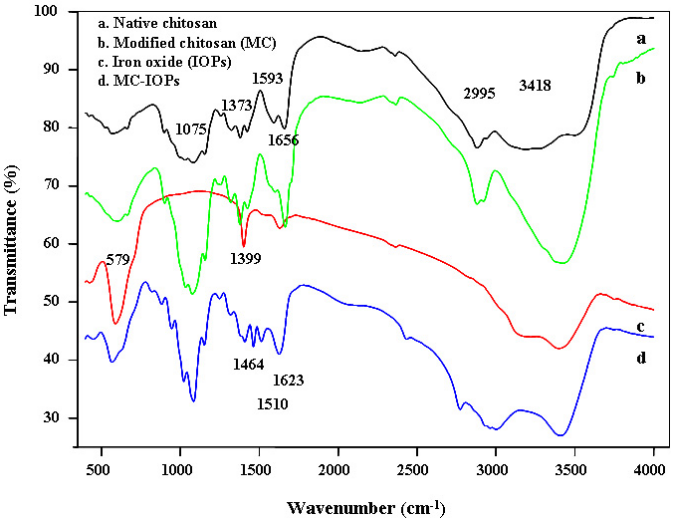
FTIR spectra of (a) pure chitosan, (b) MC, (c) pure IOPs and (d) MC-IOPs.

**Figure 3:**
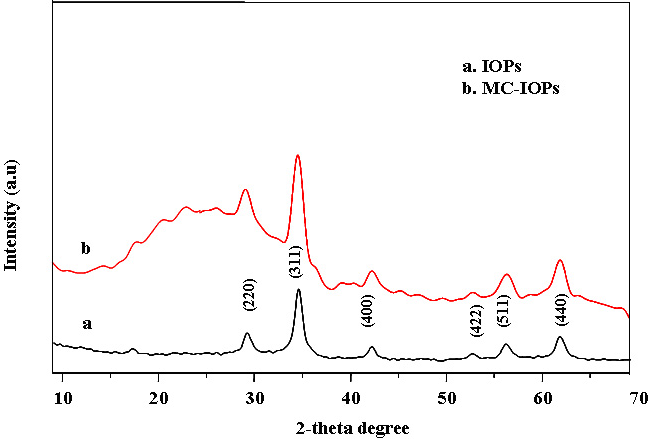
XRD pattern of (a) IOPs, (b) MC-IOPs showing only magnetite reflection.

**Figure 4:**
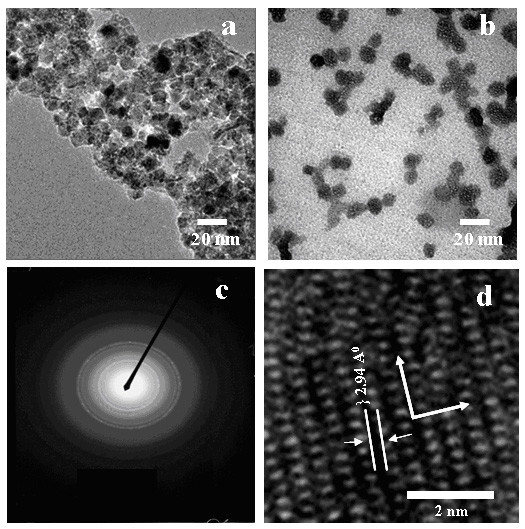
TEM images of (a) pure IOPs, (b) MC-IOPs, (c) SAD pattern of MC-IOPs and (d) HRTEM image of MC-IOPs showing a 10 nm size magnetite nanoparticle with highly polycrystalline nature.

The procedure for synthesis of IOPs, modification process of chitosan, and its detailed characterization was taken from a previously published report [9]. Briefly, Figure 2 shows the fourier transforms infrared (FT-IR) spectra of pure chitosan (a) MC (curve b), IOPs (curve c), and MC-IOPs (curve d). IOPs exhibit strong bands in the low frequency region below 800 cm-1 due to the oxide skeleton. The characteristic bands of modified chitosan, amide I, II, and III were shifted 1623, 1510 and 1464 cm-1 due to interaction with IOPs. Shifting of such amide bands from higher to lower energies indicates the attachment of IOPs with MC through nitrogen atom [8,9]. In other regions, the spectra of IOPs have weak bands. The spectrum is consistent with magnetic (Fe_3_O_4_) and the signals associated to the magnetite appear as broad features at 408.9, 571.5 and 584.5 cm^-1 ^[4]. Figure 3 shows the X-ray diffraction (XRD) pattern of IOPs matched with the magnetite (Fe_3_O_4_) phase as compared to standard XRD patterns reported elsewhere [5]. The sharp peaks which appeared approximately 2θ = 30°, 35°, 43°, 53°, 57° and 62° were due to Fe_3_O_4 _[1]. Figure 4 shows the transmission electron microscopy (TEM) images of IOPs, and MC-IOPs particles. The morphology of IOPs (Figure 4a) was seen as clustered type, which is the same morphology as reported elsewhere [10]. After modifications, morphology of IOPs was significantly dispersed with an average diameter 10 nm (average over 100 particles) (Figure 4b) in aqueous medium at pH 7.4. The average size of IOPs with and without MC was 10 and 40 nm, respectively (Figure 4a, b and table 1). Figure 4c shows the selected area diffraction (SAD) pattern of MC-IOPs, which is in exact/or in good agreement with the XRD results. The ring type SAD pattern consists of a cubic inverse spinal structure of magnetite and it indicates good crystalinity of the MC-IOPs. Figure 4d, high resolution transmission electron microscopy (HRTEM) images further support the interplaner distances of *d *= 2.94 Å which is very closed to the plane *d*_220 _= 2.97 Å of the magnetite phase in orientation. Taken together, the results of XRD, TEM and HRTEM, show that the synthesized MC-IOPs is highly crystalline as can be found in the pure magnetite phase without phase transformation after conjugation with MC, showing a successful synthesis of magnetic MC-IOPs. The results of dynamic light scattering (DLS) measurements showed a uni-model size distribution of the nanoparticles. The average sizes of the IOPs and MC-IOPs were 60 and100 nm, respectively, (Table 1, DLS measurement). In contrast to TEM measurement, DLS gave a significantly larger size in the case of both particles. The reason behind this phenomenon is obvious. The particle size measured by DLS technique is larger than that observed by TEM due to the different nature working function of the two instruments. Moreover, DLS methods differ from TEM in that it measured the hydrodynamic particle size in the dispersion medium. TEM images show the core particle size, without the contribution from the MC; since the MC layer normally collapses onto the IOPs surface when the dispersion medium is evaporated prior to imaging. It is also obvious that the thickness of the stabilizing layer (here MC), when collapsed on the surface of the IOPs, is negligible. Therefore, the difference in diameter measurements obtained by DLS and those obtained by TEM is the size of the stabilizing layer. However, this method is only valid for small particles (diameter < 200 nm), since the size of the stabilizing layer on larger particles is small relative to the experimental error inherent in DLS measurements (± 4%).

**Table 1:** Physiochemical properties of IOPs and MC-IOPs

Sample	Size (nm)		Charge (mV)	Size distribution
			
	TEM	DLS		
IOPs (Pure)	40	60	-10	Large
MC-IOPs	10	100	+20.21	Narrow

Table 1 shows the ζ-potential of IOPs and MC-IOPs. The polymer of MC being a polycation gives different +ve ζ-potential depending on the pH of the media. The ζ-potential of the MC was decreased at pH 7.4 after incorporation of IOPs. However, the ζ-potential of MC-IOPs (+20.21 mV) at physiological conditions (pH 7.4) is still acceptable for magnetofection of mammalian cells.

**Figure 5:**
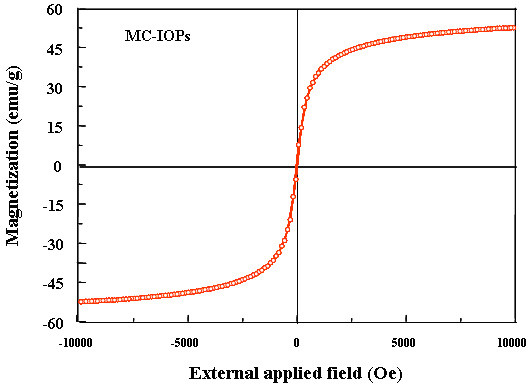
Magnetisation curve of magnetite obtained by VSM at room temperature.

Figure 5 shows the magnetic properties of magnetic nanoparticles (MC-IOPs). The synthesized MC-IOPs indicate a superparamagnetic behaviour, as evidenced by zero coercivity and remanance on the magnetization loop. A saturation magnetization of ~50 emu/g was determined for the MC-IOPs which is relatively lower than that of the bulk value of Fe_3_O_4 _(70 emu/g). The higher value of magnetization of MC-IOPs makes them very susceptible to magnetic fields, and easily separates from the solid and liquid phases.

### Biocompatibility and cellular labeling of MC-IOPs

**Figure 6:**
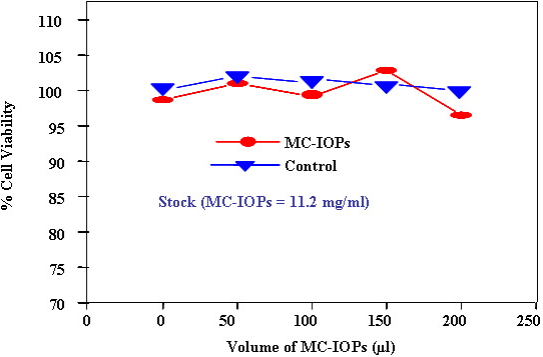
Toxicity evaluation of MC-IOPs on RAW cells by MTT assay. Different volume (50 ~200 μl) of the nanoparticles was used form the stock MC-IOPs (11.2 mg/ml).

MTT assays were performed to evaluate the cytotoxicity corresponding to the biocompatibility of the materials on RAW cell. Figure 6 shows the representative data of cyto-toxicities from three different experiments with increasing concentration of the MC-IOPs. The MC-IOPs at low concentration (<10 mg/ml) showed relatively no significant toxicity on the cells. The cell viabilities in the presence of MC-IOPs suspension ranged between 97–120% of the control in all experiments. At a maximum MC-IOPs concentration (>15 mg/ml), the mean cell viabilities of the cell lines showed about 88–97% viability compared with that of the control. Interestingly, even at high concentrations of MC-IOPs up to 100 mg/ml, which is 9~12-fold higher than the concentration required for high efficiency of intravenous injection, MC-IOPs showed no obvious negative effect on cell viability. This means that the cell viability, after exposure to different concentrations of the MC-IOPs assessed in RAW cells, apparently unaltered in the entire test dosage range from 0.05 to 0.2 mg after the 4 h of exposure, as depicted in Figure 6. The probable reason for high compatibility could be the highly biocompatible natural polymer of chitosan.

**Figure 7:**
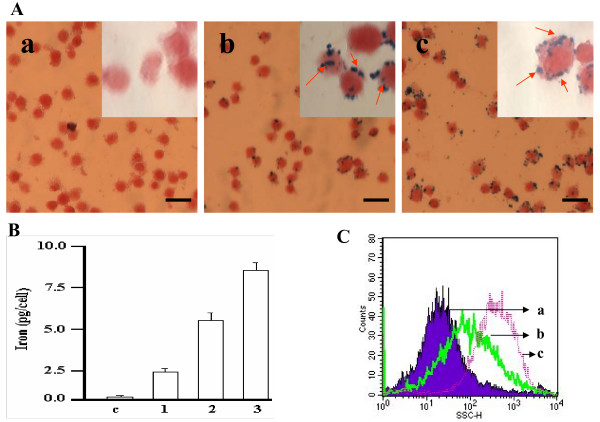
(A) Internalozation of MC-IOPs in RAW cells. Cells were cultured with different volume of MC-IOPs (11.2 mg/ml). Cytospin slides were stained with Prussian blue (iron staining) for RAW cells; (a) control cells, (b) and (c) cells incubated with 10 and 20 μl MC-IOPs for 5 h. Inset figure indicate the higher magnification and black arrow denote cell label with particles. Scale bars represent 10 μm. (B) Iron content in RAW cells. Cells were cultured with different concentration of MC-IONPs for 2 h, and incubated for 24 h with fresh medium. C, 1, 2 and 3 represent 0, 5, 10 and 20 μl of MC-IOPs from the stock 11.2 mg/ml, respectively). (C) Flow cytometry of RAW cells incubated with different concentrations of MC-IOPs as described in (A). The SSC signal (SSC-H) is increased with increased concentrations of MC-IOPs. Quantitative iron assessment was performed with a colorimetric method. Values are means of ± S.D. of iron content per single RAW cells (pg).

Semiqantitative microscopic analysis showed that the MC-IOPs were incorporated by RAW cells in a concentration and time dependent manner, Figure 7. At the low concentration of the MC-IOPs, only few cells showed intracytoplasmatic Prussian blue positive particles, Figure 7A(b). At a high concentration of the MC-IOPs, virtually all cells contained several Prusssian blue-stained, Figure 7A(c), and no cellular loss or damage was observed. Whatever the MC-IOPs concentration, the RAW cells stated internalize the MC-IOPs after 30 min, Figure 7A(b) and 7(c), inset and reached a plateau after 3~4 h, Figure 7A(c). Furthermore, colorimetric quantitative method was used to determine and confirm dose-dependent nanoparticle internalization by observing the RAW cell microscopically (Figure 7B). At our optimal experimental setting based on the morphological observation (10~20 MC-IOPs for 2 h incubation time, Figure 7A(b) and 7(c), the macrophages RAW cells contained an average of 8.53 ± 0.31 pg (iron/cell), Figure 7B. Similarly, Figure 7C demonstrates the side scattering (SSC) distribution of cell shifted with increasing concentration of the MC-IOPs, which means an increase in granularity with increasing MC-IOPs concentration. This finding is important because we suspect the phagocytosed MC-IOPs became endosomes and thereby increased the granularity found in flow cytometry. These results further support the semiquantitative microscopic analysis (Prussian blue-stained).

### Magnetic resonance (MR) study of MC-IOPs

**Figure 8:**
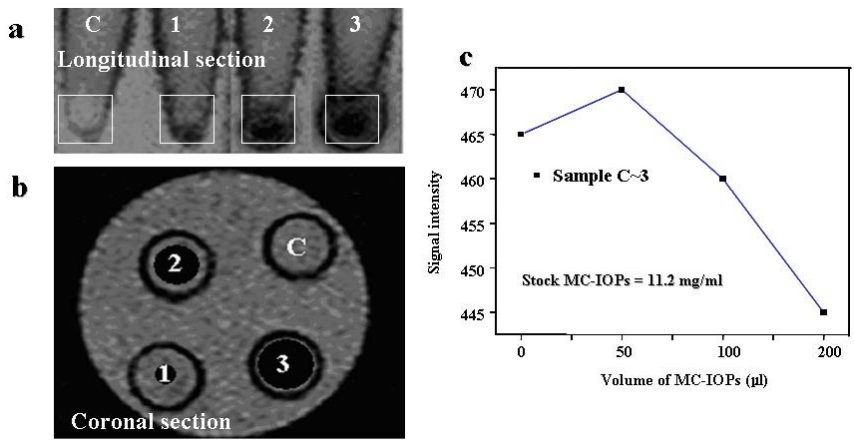
T2 weighted MR images of a representive RAW cells incubated with different volume of MC-IOPs (11.2 mg/ml) for 5 h, (a) longitudinal section, (b) coronal section and (c) signal intensity of sample c to 3 (lane c~3; control, 50, 100 and 200 μl MC-IOPs, respectively).

Figure 8a and 8c, illustrated the signal contrast enhancement performance of the MC-IOPs incubated with RAW cells evaluated in clicical MR imager. This typical array image of the RAW cells, with a concentration gradient of the MC-IOPs in an incubated media solution, is taken by T2 MR sequence. Under T2 weighted pulse sequence evaluation, the signal of each cell pellet was measured as shown in Figure 8a and 8b. The image was further converted into signal intensity by the provided image analysis tool for quantitative measurements. Figure 8c demonstrated the signal difference between cells with and without MC-IOPs incubation. These results clearly indicate that the signal intensity gradually dropped in the iron concentration above 0.1 mg/ml which was in good agreement to the results reported elsewhere [11].

## Conclusion

MC-IOPs synthesized by simple precipitation method showed highly crystalline, superparamagnetic behavior. It also displayed high stability, nontoxicity, enhancement of MR images and the potential endocytose the macrophage cell line. From above preliminary results, we conclude that MC-IOPs could be a better candidate for MR contrast medium.

## Experimental methods

### Materials

Iron (III) chloride hexahydrate (FeCl_3 _6H_2_O) pure granulated, 99%, iron (II) chloride tetrahydrate (FeCl_2 _4H_2_O) 99+%, and ammonium hydroxide (14.8 M) were purchased from Fisher Scientific (Pittsburgh, PA). Deionized water purged with nitrogen gas was used in all the steps involved in the synthesis and formulation of iron oxide nanoparticles. Chitosan-100 [viscosity average molecular weight, Mv = 1.3 × 10^6^, degree of deacetylation (fraction of free amino group) 78%] was purchased from Wako Pure Chemical Industries, Ltd., Japan.

### Synthesis of iron oxide nanoparticles (IOPs)

Aqueous solutions of 0.1 M Fe(III) (30 mL) and 0.1 M Fe(II) (15 mL) were mixed, and 3 mL of 5 M ammonia solution was added dropwise over 1 min while stirring on a magnetic stir plate. The stirring continued for 20 min under a nitrogen-gas atmosphere. The particles obtained were washed 3 times using ultracentrifugation (25000 × g for 20 min at 4°C) with nitrogen purged water. The iron oxide nanoparticle yield, determined by weighing of the lyophilized sample of the preparation, was 304 mg.

### Modification of chitosan (MC)

The modification process of chitosan was taken from a previously described report [7-9]. Briefly, a mixture of chitosan-100 (0.83 g) and 1.0% aqueous acetic acid (100 ml) was stirred for 24 h to ensure total solubility. The pH was adjusted to 7.0 by slow addition of 0.1 M of NaOH with strong agitation, yielding gel slurry. After addition of 0.02 M of fatty acyl chloride (hexanoyl chloride, FW = 134.61, *d *= 0.978 g/ml), the resultant solution was diluted 11 times with de-ionized water. After 6 h of continuous stirring, the solution was neutralized (pH 6.8–7.0) by 0.1 M of NaOH and precipitated with acetone. The precipitate, collected by filtration, was washed at 50–60°C with an excess of methanol and decanted. The washing was repeated 4 times to eliminate free fatty acids. Finally, the products were dried under vacuum for 3 days at room temperature. The chemical structure of native and modified chitosan is shown in Figure 1.

### Stabilization of iron oxide nanoparticles (MC-IOPs)

Polymer (5.0 ml of 0.33% of *N*-hexanoyl chitosan solutions or MC) was added to the dispersion of the nanoparticles (100 mg) (the dispersion was cooled to room temperature but not lyophilized) and stirred overnight in a closed container to minimize exposure to atmospheric oxygen to prevent oxidation of the IOPs. These particles were washed with nitrogen purged water to remove soluble salts and excess polymer. Particles were separated by ultracentrifugation at 30000 rpm (Optima LE-80K, Beckman, Palo Alta, CA) using a fixed angle rotor (50.2 Ti) for 30 min at 10°C. The supernatant was discarded, and the sediment was redispersed in 15 mL of triply distilled water by sonication in a water-bath sonicator (FS- 30, Fisher Scientific) for 10 min. The suspension was centrifuged as above, and the sediment was washed three times with triply distilled water. Nanoparticles were resuspended in triply distilled water by sonication as above for 20 min and centrifuged at 1000 rpm for 20 min at 7–11°C to remove any large aggregates. The supernatant containing MC-IOPs was collected and re-diluted in phosphate buffer at pH 7.4.

### Structural characterization of MC-IOPs

FT-IR spectra were recorded at RT using a Perkin-Elmer spectrometer, model 2000. The FT-IR spectrometer was linked to a personal computer loaded with the IRDM (IR Data Manager) program to process the recorded spectra. The specimens were pressed into small discs using a spectroscopically pure KBr matrix. FT-IR measurements were checked by the X-ray diffraction of isolated precipitates. XRD (APD-10, Philips, Netherlands) was performed to identify the structure of the MC-IOPs using Cu K alpha radiation (*λ *= 1.54056 Å) between 20° and 90° (2*θ*) at 27°C.

### Particle size, morphology and ξ-potential analysis of MC-IOPs

The size and morphology of IOPs and MC-IOPs were observed by TEM (JEM-1230, JEOL, Japan) and HRTEM (QUANTA 200F, FEI, USA). The sample for TEM analysis was obtained by placing a drop of IOPs and MC-IOPs suspension diluted by distilled water onto a copper grid without any staining, and drying it in air at room temperature. The average hydrodynamic diameter and the *ξ*-potential of IOPs and MC-IOPs were determined by DLS and ELS (Zetasizer ZEN 3600, Malvern, UK), respectively. All DLS measurements were done with an angle detection of 90° at 25°C after diluting the dispersion to an appropriate volume with water. The results were the mean values of two experiments using the same sample.

### Magnetic property of MC-IOPs

Magnetic measurement was done using a SQUID magnetometer (MPMSXL-7, Quantum Design, USA). Magnetization curves were recorded for a suspension and solid sample of MC-IOPs at 27°C with an applied magnetic field up to 10,000 Oe.

### Evaluation of cytotoxicity

Evaluation of the cytotoxicity was performed by the MTT assay in RAW cells (mouse macrophases cell lines). Briefly, RAW cells suspensions containing 1 × 10^4 ^cell/well in DMEM containing 10% FBS were distributed in a 96-well plates, and incubated in a humidified atmosphere containing 5% CO_2 _at 37°C for 24 h [12,13]. The cytotoxicity of MC-IOPs was evaluated in comparison with control cells. Cells were incubated for additional 24 h after the addition of defined concentration of MC-IOPs. The mixture was replaced with fresh medium containing 10% FBS. Then, 20 μl of MTT solution (5 mg/ml in 1 × PBS) were added to each well. The plate was incubated for an additional 4 h at 37°C. Next, MTT-containing medium was aspirated off and 150 μl of DMSO were added to dissolve the crystals formed by living cells. Absorbance was measured at 490 nm, using a microplate reader (ELX 800; BIO-TEK Instruments, Inc.). The cell viability (%) was calculated according to the following equation:

Cell viability (%) = [OD 490(sample)/OD 490(control)] × 100

### Cellular uptake of MC-IOPs

To test cell up take study, RAW cells were prepared and incubated at a concentration of 1 × 10^6 ^cells/ml with 5, 10 and 20 μl MC-IOPs (11.2 mg/ml stock) for 2 h, then incubated with fresh medium overnight. The cells were harvested and measured by flow cytometry using SSC signal. Similarly harvested RAW cells were further used for Prussain blue staining using K_4 _[Fe(CN)_6_] reagents. Iron determination was performed by colorimetric determination method.

### Magnetic resonance (MR) study of MC-IOPs

For MR study, MC-IONPs were incubated with RAW cells at different concentration for 24 h. The cells were harvested and washed three times and centrifuged at the cell number 1 × 10^3^. The cell plates were scanned using 1.5T MR system. Under T2 weighted MR images of MC-IONPs were obtained with 1.5T MR system (Medius Co. Korea, Model Magnum1.5T) by using a spin echo technique. The differences between MR images of cells with and without MC-IONPs incubation were compared.

## Abbreviations

MC- Hexanoyl chloride modified chitosan or simply modified chitosan;

MRI: Magnetic resonance imaging;

IOPs: Iron oxide nanoparticles).

## Competing interests

The author(s) declare that they have no competing interests.

## Authors' contributions

SRB did almost all of the experiments and data analysis in the laboratory, RBKc helped with polymer modification, MS helped with the molecular biology work, SYK and MSK coordinated experiments, PHH provided important advice for the experiments and coordinated experiments with radiology department, GHC helped with MR measurements and HYK provided important advice and financial support. All authors read and approved the final manuscript.
